# Supplementary figures and images for: Prognostic and therapeutic insights into colorectal carcinoma through immunogenic cell death gene profiling
Source: PeerJ. 2024 Jun 24;12:e17629. doi: 10.7717/peerj.17629 (PMC11210462; doi:10.7717/peerj.17629)

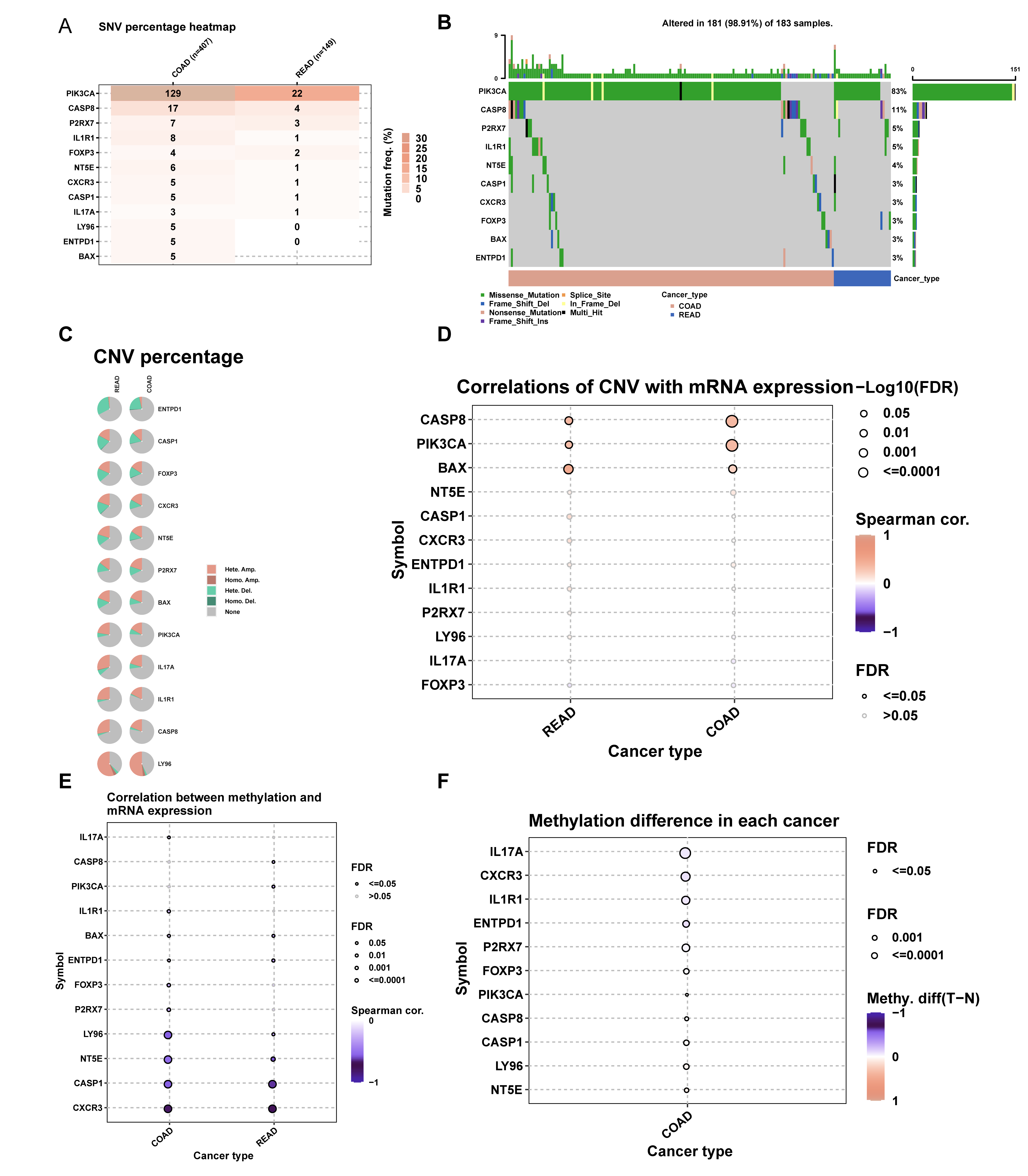

Supplement: Supplemental Information 1 — (A) Cox regression of ICD signature genes. Forest plot showing hazard ratios (HRs) for 25 key genes with p < 0.00001. (B) SNV heatmap illustrate SNV percentages in ICD signature genes. (C) Genetic alterations in patients. 181 of 183 patients showed changes in these genes. (D) CNV mutation frequency. Pie plots summarizing CNV in ICD genes. (E) CNV and gene correlation. (F) Methylation and gene correlation. (G) Methylation differences. Compares ICD gene methylation in COAD vs. normal samples. [file peerj-12-17629-s001.png]

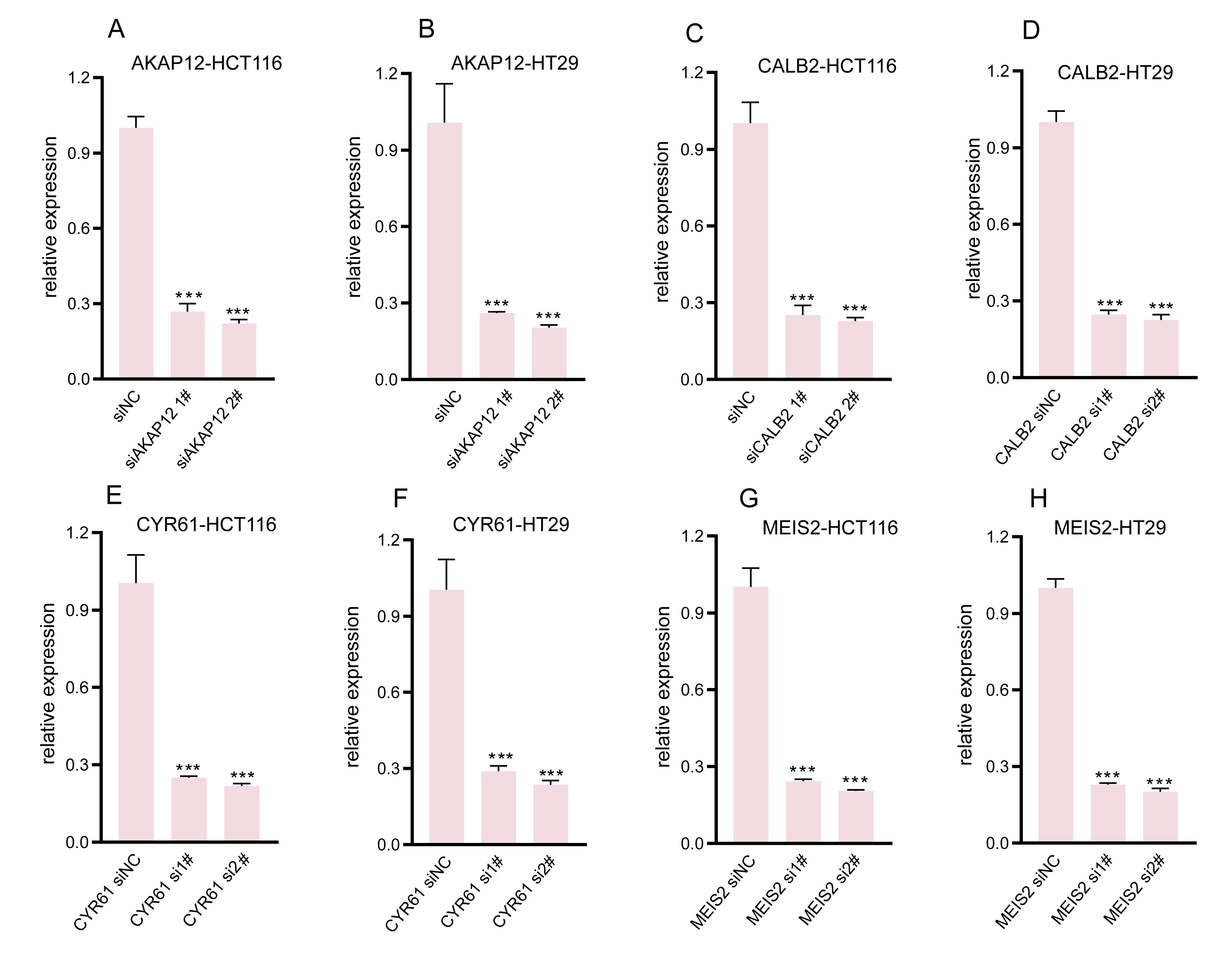

Supplement: Supplemental Information 2 — Quantitative RT-PCR analysis assessing mRNA levels of AKAP12 (A, B), CALB2 (C, D), CYR61 (E, F), and MEIS2 (G, H) post-transfection in HCT116 and HT29 cells. Significant mRNA expression reduction observed in all cases (P<0.001). [file peerj-12-17629-s002.png]
