# Supplementary material for: Prognostic and therapeutic insights into colorectal carcinoma through immunogenic cell death gene profiling
Source: PeerJ. 2024 Jun 24;12:e17629. doi: 10.7717/peerj.17629 (PMC11210462; doi:10.7717/peerj.17629)
Supplement: Supplemental Information 3 [file peerj-12-17629-s003.docx]

| Gene | Sense | Antisense |
| --- | --- | --- |
| AKAP12 | GAGATGGCTACTAAGTCAGCGG | CAGTGGGTTGTGTTAGCTCTTC |
| CALB2 | ACTTTGACGCAGACGGAAATG | GAAGTTCTCTTCGGTTGGCAG |
| CYR61 | CTCGCCTTAGTCGTCACCC | CGCCGAAGTTGCATTCCAG |
| MEIS2 | GAAAAGGTCCACGAACTGTGC | CTTTCATCAATGACGAGGTCGAT |
| CD274 | TGGCATTTGCTGAACGCATTT | TGCAGCCAGGTCTAATTGTTTT |
| Gene | siRNA 1# | siRNA 2# |
| AKAP12 | GATGAAGTGGAAGCTTCAGAGAAGA | GACACAGAAGAAGACGGAAAGGCAG |
| CALB2 | CAGTTCCTGGAAATATGGAAGCACT | CGCAGACGGAAATGGGTATATTGAA |
| CYR61 | CGAGGTGGAGTTGACGAGAAACAAT | CAAACAACTTCATGGTCCCAGTGCT |
| MEIS2 | TGGGCACCCGTTGTTTCCTCTGTTA | CAGAGCTGGACAATTTGATGATACA |
